# Bis[5-oxo-4,5-dihydro-8*H*-2-azonia-4,8,9-trizabicyclo[4.3.0]nona-2,6,9(1)-triene] sulfate

**DOI:** 10.1107/S1600536808038026

**Published:** 2008-11-22

**Authors:** Nittala V. Ravindra, Gopal M. Panpalia, Jagarlapudi A. R. P. Sarma

**Affiliations:** a Birla Institute of Technology, Department of Pharmaceutical Sciences, Mesra, Ranchi 835 215, India; b GVK Biosciences Private Limited, S-1, Phase-1 Technocrats Industrial Estate, Balanagar, Hyderabad 500 037, India

## Abstract

In the crystal structure of the title compound, 2C_5_H_5_N_4_O^+^·SO_4_^2−^, N—H⋯O hydrogen bonds assemble the mol­ecules into a two-dimensional network structure parallel to the *cb* plane. The S atom of the sulfate ion lies on a special position on a twofold axis.

## Related literature

For general background, see: Elion *et al.* (1962 ▶); Rundles *et al.* (1966 ▶). For related structures, see: Prusiner & Sundaralingam (1972 ▶); Gadret *et al.* (1974 ▶); Sheldrick & Bell (1987 ▶); Singh & Pedersen (1993 ▶).
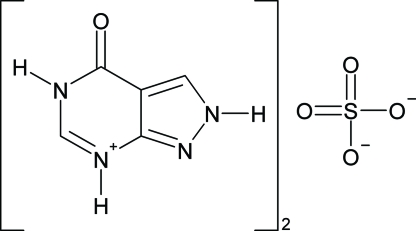


## Experimental

### 

#### Crystal data

2C_5_H_5_N_4_O^+^·SO_4_^2−^
*M* *_r_* = 370.32
Monoclinic, 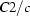

*a* = 12.337 (3) Å
*b* = 10.054 (2) Å
*c* = 11.064 (2) Å
β = 102.42 (3)°
*V* = 1340.2 (5) Å^3^
*Z* = 4
Mo *K*α radiation
μ = 0.30 mm^−1^
*T* = 298 (2) K
0.20 × 0.20 × 0.10 mm

#### Data collection

Bruker SMART CCD area-detector diffractometer
Absorption correction: none
3561 measured reflections
1323 independent reflections
1252 reflections with *I* > 2σ(*I*)
*R*_int_ = 0.017

#### Refinement

*R*[*F* ^2^ > 2σ(*F* ^2^)] = 0.035
*wR*(*F* ^2^) = 0.096
*S* = 1.05
1323 reflections
132 parameters
All H-atom parameters refined
Δρ_max_ = 0.32 e Å^−3^
Δρ_min_ = −0.38 e Å^−3^

### 

Data collection: *SMART* (Bruker, 1997 ▶); cell refinement: *SAINT* (Bruker, 1997 ▶); data reduction: *SAINT*; program(s) used to solve structure: *SHELXS97* (Sheldrick, 2008 ▶); program(s) used to refine structure: *SHELXL97* (Sheldrick, 2008 ▶); molecular graphics: *SHELXTL* (Sheldrick, 2008 ▶); software used to prepare material for publication: *SHELXTL*.

## Supplementary Material

Crystal structure: contains datablocks I, global. DOI: 10.1107/S1600536808038026/gw2054sup1.cif

Structure factors: contains datablocks I. DOI: 10.1107/S1600536808038026/gw2054Isup2.hkl

Additional supplementary materials:  crystallographic information; 3D view; checkCIF report

## Figures and Tables

**Table 1:** Hydrogen-bond geometry (Å, °)

*D*—H⋯*A*	*D*—H	H⋯*A*	*D*⋯*A*	*D*—H⋯*A*
N2—H3⋯O3^i^	0.936 (10)	1.698 (14)	2.628 (2)	173 (3)
N3—H5⋯O2	0.819 (10)	1.957 (18)	2.753 (3)	164 (3)
N1—H1⋯O3^ii^	0.906 (10)	1.838 (13)	2.716 (3)	163 (3)

Symmetry codes: (i) 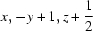; (ii) 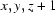.

**Fig. 1.**
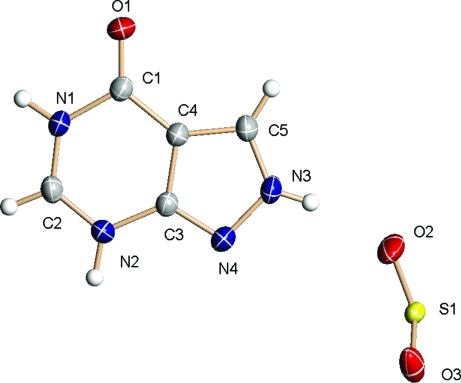
The molecular structure of (I), with atom labels and 50% probability displacement ellipsoids for non-H atoms.

**Fig. 2.**
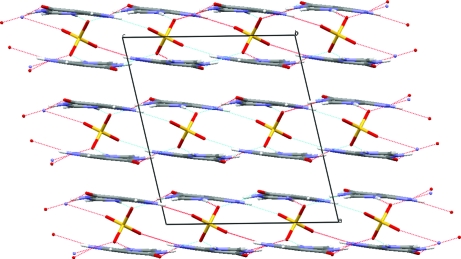
The packing of (I), viewed down the b axis, showing the layers of molecules and sulfate molecules connected by N—H···O hydrogen bonds (dashed lines).

## supplementary crystallographic information

### Comment

Allopurinol is known to be a potent inhibitor of xanthine oxidase and is used extensively for treatment of gout (Rundles *et al.*, 1966). It is used in conjunction with anticancer drugs which impede RNA biosynthesis and as adjunct therapy in conjunction with 6-mecaptopurine in treatment of leukemia (Elion *et al.*, 1962). As part of our interest in salts and co-crystals of drugs, we have investigated the crystal structure of Allopurinol sulfate, (I) (Fig. 1). The least-squares plane of the six-membered ring makes an angle of 5.79 (11)° with the least-squares planes of the five-membered ring of the purine ring. The N3 nitrogen is protonated similar to the reported structure of chloride salt (Sheldrick & Bell, 1987). The molecules are linked *via* N—H···O hydrogen bonds,forming two-dimensional infinite chains along the *cb* plane. These sheets are linked together by the sulfate molecules which act as acceptors of H atoms, assembling the molecules in an infinite two-dimensional network (Fig. 2).

### Experimental

Allopurinol (0.1361 g, 1 mmol) and Sulfuric acid (0.27 ml, 5 mmol) were added to Dimethylformamide. The mixture was stirred and heated on a water bath. After solution was complete, it was filtered and allowed to evaporate at room temperature. Colorless plate like crystals, suitable for *x*-ray analysis grew over a period of two days when the solution was exposed to air.

### Refinement

Atoms H1, H3 and H5 on N1, N2 and N3 respectively were located in difference Fourier maps and refined isotropically. All other H atoms were found in difference map but then placed in calculated positions with C—H = 0.93 Å and *U*_iso_(H) = 1.2Ueq(C) for all aromatic H atoms.

### Crystal data

**Table:**

2C_5_H_5_N_4_O^+^·S_1_O_4_^2–^	*F*_000_ = 760
*M**_r_* = 370.32	*D*_x_ = 1.835 Mg m^−^^3^
Monoclinic, *C*2/*c*	Mo *K*α radiation λ = 0.71073 Å
Hall symbol: -C 2yc	Cell parameters from 1323 reflections
*a* = 12.337 (3) Å	θ = 2.6–26.1º
*b* = 10.054 (2) Å	µ = 0.30 mm^−^^1^
*c* = 11.064 (2) Å	*T* = 298 (2) K
β = 102.42 (3)º	Plate, colourless
*V* = 1340.2 (5) Å^3^	0.20 × 0.20 × 0.10 mm
*Z* = 4	

### Data collection

**Table:**

Bruker SMART CCD area-detector diffractometer	1252 reflections with *I* > 2σ(*I*)
Radiation source: fine-focus sealed tube	*R*_int_ = 0.017
Monochromator: graphite	θ_max_ = 26.1º
*T* = 298(2) K	θ_min_ = 2.6º
φ and ω scans	*h* = −15→11
Absorption correction: none	*k* = −10→12
3561 measured reflections	*l* = −13→13
1323 independent reflections	

### Refinement

**Table:**

Refinement on *F*^2^	Hydrogen site location: inferred from neighbouring sites
Least-squares matrix: full	All H-atom parameters refined
*R*[*F*^2^ > 2σ(*F*^2^)] = 0.035	*w* = 1/[σ^2^(*F*_o_^2^) + (0.0621*P*)^2^ + 0.9856*P*] where *P* = (*F*_o_^2^ + 2*F*_c_^2^)/3
*wR*(*F*^2^) = 0.096	(Δ/σ)_max_ < 0.001
*S* = 1.05	Δρ_max_ = 0.32 e Å^−^^3^
1323 reflections	Δρ_min_ = −0.38 e Å^−^^3^
132 parameters	Extinction correction: SHELXL97 (Sheldrick, 2008), Fc^*^=kFc[1+0.001xFc^2^λ^3^/sin(2θ)]^-1/4^
Primary atom site location: structure-invariant direct methods	Extinction coefficient: 0.0068 (13)
Secondary atom site location: difference Fourier map	

### Special details

**Table:**

Geometry. All e.s.d.'s (except the e.s.d. in the dihedral angle between two l.s. planes) are estimated using the full covariance matrix. The cell e.s.d.'s are taken into account individually in the estimation of e.s.d.'s in distances, angles and torsion angles; correlations between e.s.d.'s in cell parameters are only used when they are defined by crystal symmetry. An approximate (isotropic) treatment of cell e.s.d.'s is used for estimating e.s.d.'s involving l.s. planes.
Refinement. Refinement of *F*^2^ against ALL reflections. The weighted *R*-factor *wR* and goodness of fit *S* are based on *F*^2^, conventional *R*-factors *R* are based on *F*, with *F* set to zero for negative *F*^2^. The threshold expression of *F*^2^ > σ(*F*^2^) is used only for calculating *R*-factors(gt) *etc*. and is not relevant to the choice of reflections for refinement. *R*-factors based on *F*^2^ are statistically about twice as large as those based on *F*, and *R*- factors based on ALL data will be even larger.

### Fractional atomic coordinates and isotropic or equivalent isotropic displacement parameters (Å^2^)

**Table:**

	*x*	*y*	*z*	*U*_iso_*/*U*_eq_	
S1	0.5000	0.24338 (5)	0.2500	0.0248 (2)	
O3	0.41241 (12)	0.33107 (12)	0.17777 (11)	0.0458 (4)	
O2	0.45510 (13)	0.16122 (14)	0.33449 (12)	0.0472 (4)	
O1	0.32972 (10)	0.07703 (11)	0.91973 (10)	0.0354 (3)	
N1	0.35100 (11)	0.30200 (13)	0.92837 (12)	0.0279 (3)	
C4	0.35629 (12)	0.19500 (15)	0.73986 (13)	0.0244 (3)	
N3	0.38403 (11)	0.18821 (14)	0.55266 (13)	0.0289 (3)	
N4	0.38369 (11)	0.32129 (13)	0.57759 (12)	0.0290 (3)	
N2	0.36693 (11)	0.43601 (13)	0.76273 (12)	0.0281 (3)	
C1	0.34391 (12)	0.17773 (15)	0.86560 (14)	0.0254 (3)	
C3	0.36700 (12)	0.32242 (14)	0.69190 (14)	0.0245 (3)	
C5	0.36919 (13)	0.11072 (16)	0.64455 (14)	0.0275 (3)	
C2	0.36240 (13)	0.41996 (16)	0.87928 (14)	0.0287 (4)	
H2	0.3674 (16)	0.4935 (19)	0.9297 (18)	0.033 (5)*	
H4	0.3710 (16)	0.018 (2)	0.6442 (18)	0.036 (5)*	
H3	0.3781 (18)	0.522 (2)	0.735 (2)	0.049 (6)*	
H5	0.3967 (18)	0.168 (2)	0.485 (2)	0.045 (6)*	
H1	0.3606 (18)	0.299 (2)	1.012 (2)	0.044 (5)*	

### Atomic displacement parameters (Å^2^)

**Table:**

	*U*^11^	*U*^22^	*U*^33^	*U*^12^	*U*^13^	*U*^23^
S1	0.0361 (3)	0.0221 (3)	0.0175 (3)	0.000	0.0085 (2)	0.000
O3	0.0595 (8)	0.0395 (7)	0.0306 (6)	0.0168 (6)	−0.0078 (6)	−0.0102 (5)
O2	0.0712 (9)	0.0445 (8)	0.0339 (7)	−0.0155 (7)	0.0293 (7)	−0.0026 (6)
O1	0.0502 (7)	0.0289 (6)	0.0290 (6)	−0.0007 (5)	0.0131 (5)	0.0064 (5)
N1	0.0360 (7)	0.0293 (7)	0.0196 (7)	0.0007 (5)	0.0085 (5)	−0.0018 (5)
C4	0.0280 (7)	0.0244 (8)	0.0212 (7)	−0.0003 (5)	0.0063 (6)	−0.0002 (5)
N3	0.0348 (7)	0.0323 (8)	0.0208 (6)	0.0000 (5)	0.0088 (5)	−0.0051 (5)
N4	0.0354 (7)	0.0306 (7)	0.0220 (7)	0.0002 (5)	0.0081 (5)	0.0016 (5)
N2	0.0372 (7)	0.0218 (7)	0.0256 (7)	0.0006 (5)	0.0075 (5)	0.0001 (5)
C1	0.0268 (7)	0.0279 (8)	0.0220 (7)	0.0008 (6)	0.0065 (6)	−0.0001 (5)
C3	0.0267 (7)	0.0259 (8)	0.0209 (7)	0.0008 (5)	0.0051 (6)	−0.0011 (5)
C5	0.0320 (8)	0.0264 (8)	0.0246 (7)	−0.0005 (6)	0.0071 (6)	−0.0044 (6)
C2	0.0333 (8)	0.0273 (8)	0.0251 (7)	0.0017 (6)	0.0056 (6)	−0.0052 (6)

### Geometric parameters (Å, °)

**Table:**

S1—O2^i^	1.4442 (12)	C4—C1	1.442 (2)
S1—O2	1.4443 (12)	N3—C5	1.324 (2)
S1—O3	1.4873 (13)	N3—N4	1.366 (2)
S1—O3^i^	1.4873 (13)	N3—H5	0.82 (2)
O1—C1	1.2081 (18)	N4—C3	1.324 (2)
N1—C2	1.324 (2)	N2—C2	1.313 (2)
N1—C1	1.423 (2)	N2—C3	1.3851 (19)
N1—H1	0.91 (2)	N2—H3	0.94 (2)
C4—C5	1.388 (2)	C5—H4	0.94 (2)
C4—C3	1.404 (2)	C2—H2	0.92 (2)
			
O2^i^—S1—O2	110.23 (11)	C2—N2—C3	117.38 (13)
O2^i^—S1—O3	109.07 (8)	C2—N2—H3	118.7 (13)
O2—S1—O3	110.57 (9)	C3—N2—H3	123.5 (13)
O2^i^—S1—O3^i^	110.57 (9)	O1—C1—N1	119.61 (14)
O2—S1—O3^i^	109.07 (8)	O1—C1—C4	129.47 (14)
O3—S1—O3^i^	107.30 (11)	N1—C1—C4	110.91 (13)
C2—N1—C1	125.94 (13)	N4—C3—N2	124.51 (14)
C2—N1—H1	116.4 (14)	N4—C3—C4	113.56 (13)
C1—N1—H1	116.7 (14)	N2—C3—C4	121.80 (13)
C5—C4—C3	103.56 (13)	N3—C5—C4	106.34 (14)
C5—C4—C1	135.43 (14)	N3—C5—H4	125.2 (12)
C3—C4—C1	120.84 (13)	C4—C5—H4	128.4 (12)
C5—N3—N4	114.47 (13)	N2—C2—N1	122.93 (14)
C5—N3—H5	129.3 (15)	N2—C2—H2	119.1 (12)
N4—N3—H5	116.1 (15)	N1—C2—H2	118.0 (12)
C3—N4—N3	102.07 (12)		

Symmetry codes: (i) −*x*+1, *y*, −*z*+1/2.

### Hydrogen-bond geometry (Å, °)

**Table:**

*D*—H···*A*	*D*—H	H···*A*	*D*···*A*	*D*—H···*A*
N2—H3···O3^ii^	0.936 (10)	1.698 (14)	2.628 (2)	173 (3)
N3—H5···O2	0.819 (10)	1.957 (18)	2.753 (3)	164 (3)
N1—H1···O3^iii^	0.906 (10)	1.838 (13)	2.716 (3)	163 (3)

Symmetry codes: (ii) *x*, −*y*+1, *z*+1/2; (iii) *x*, *y*, *z*+1.
